# Exploration of non-hierarchical classification methods combined with linkage analysis to identify loci influencing clusters of co-regulated transcripts

**DOI:** 10.1186/1753-6561-1-s1-s48

**Published:** 2007-12-18

**Authors:** Alka Malhotra, Helen C Looker, Robert L Hanson

**Affiliations:** 1 Genetic Basis of Human Disease Division, Translational Genomics Research Institute, 445 North Fifth Street, Phoenix, Arizona 85004, USA; 2 Diabetes Epidemiology and Clinical Research Section, National Institute of Diabetes & Digestive & Kidney Diseases, 1550 East Indian School Road, Phoenix, Arizona 85014, USA

## Abstract

Extensive studies have been performed to analyze variation in gene expression data by using multistage approaches, including a combination of microarray and linkage analysis. Such a method was recently used in the analysis of normal variation in gene expression by Cheung et al. (*Nat. Genet*. 2003, **33**: 422–425) and Morley et al. (*Nature *2004, **430**: 743–747). Using these data, we also explored a multistage method by first performing non-hierarchical clustering for 3554 genes, which identified 114 clusters with number of genes ranging from 2 to 113. Heritabilities of the first principal component of each cluster were then estimated and 29 highly heritable clusters (i.e., *h*^2 ^> 0.35) were further analyzed using variance components linkage analysis. The highest LOD score was observed on chromosome 1 (LOD = 5.36, 111.71 cM) for a cluster containing two genes [*glutathione S-transferase M1 *(*GSTM1*) and *glutathione S-transferase M2 *(*GSTM2*)] that are both located on chromosome 1p13.3. These results show the method followed in our analysis of performing cluster analysis followed by linkage analysis is another useful approach to identify chromosomal locations for genes affecting expression levels of multiple transcripts.

## Background

Data mining of large genetic data sets has been a primary focus in recent years. Although a large number of methods exist [1], novel approaches combining currently available and/or new methods are still needed to achieve the end goal of gene identification. In a recent study by Morley et al. [2], a genome scan was performed for variation in gene expression levels. In regions showing evidence for linkage with expression of multiple genes, hierarchical cluster analysis was performed to identify genes with potentially common genetic regulatory mechanisms. The data used in their analysis were contributed to the Genetic Analysis Workshop 15 (GAW15) as Problem 1. We took a different multistage approach to elucidate the genetics underlying variation of gene expression levels in which cluster analysis was performed prior to linkage analysis. Specifically, our approach involved the following steps: 1) identify clusters by performing non-hierarchical clustering for 3554 genes, 2) estimate the first principal component (PC1) for each cluster, 3) estimate heritability for PC1 of each cluster, and 4) perform variance-components linkage analysis for highly heritable clusters. While some investigators have used cluster analysis to identify potential pathways among transcripts linked to a region of interest, to our knowledge, this is the first study exploring the use of cluster analysis to identify pathways *de novo *prior to linkage analysis.

## Methods

### Phenotypic and genotypic data

Data for Problem 1 were used in this study. Briefly, gene expression data were measured for 3554 genes (using Affymetrix Human Focus Arrays) in 194 individuals (99 males and 95 females) from 14 families originally collected for the CEPH (Centre d'Etude du Polymorphisme Humain) study [3]. Family size ranged from 13 to 14 individuals. Genotypes from 2819 autosomal single-nucleotide polymorphism (SNP) markers generated by the SNP Consortium (also provided as part of Problem 1) were used for linkage analyses.

### Non-hierarchical clustering

The k-means algorithm [4], as implemented in SAS PROC FASTCLUS (SAS Institute, Cary, NC), was used to assign genes to clusters based on similarity of expression levels within individuals. Prior to analysis, expression levels for all 3554 transcripts were normalized with an inverse Gaussian transformation of the ranks; with 194 individuals the normalized expression levels ranged from -2.72 to 2.72. These normalized expression scores were taken as the coordinates in *n*-dimensional space (where *n *= 194) for each transcript. The k-means algorithm was then applied to assign transcripts to clusters in a way that minimizes the within-cluster distance and maximizes the between-cluster distance. Thus, genes within clusters are those which show similar normalized expression levels within an individual. The algorithm requires an operational cluster definition, which can be obtained, for example, by specification of the maximum number of clusters allowed and the minimum cluster size. For the present analyses we used a minimum cluster size of two because we were interested in the potential genetic regulation of multiple transcripts, and a maximum number of clusters of 3000, because this appeared to generate the largest number of clusters. With these parameters, 114 clusters were identified with cluster sizes ranging from 2 to 113.

### Principal-components analysis and estimation of heritability

For each cluster, the first principal component (PC1) among all transcripts in the cluster was taken as the linear combination of normalized scores that accounted for the largest proportion of the total variance [5]. The PC1 for each cluster was then used in subsequent analyses of heritability and linkage; to ensure that the required assumption of multivariate normality was met, the inverse Gaussian transformation of the ranks for each PC1 was taken prior to these analyses. Heritability of PC1 for each cluster was estimated using variance-components methods [6]. The trait variance was partitioned into a heritable component (σ^2^_G_) and an environmental component (σ^2^_E_). The variance-covariance matrix for individuals in a given pedigree is Φσ^2^_G _+ Iσ^2^_E_, where Φ is a matrix of the expected proportion of alleles shared identical by descent among family members and I is an identity matrix. Heritability was taken as *h*^2 ^= σ^2^_G_/(σ^2^_G _+ σ^2^_E_).

### Linkage analysis

Clusters with heritability estimates of PC1 ≥ 0.35 were analyzed by variance components linkage methods, as implemented in the Merlin software [7]. Twenty-nine clusters were analyzed in this manner. For the map for SNP markers we used the approximation of 1 Mb = ~1 cM. In the assessment of statistical significance of the linkage results, all LOD scores > 3 (*p *< 0.0001) were considered to have significant trait-wise linkage. To further correct for the analysis of 29 different traits in the assessment of experiment-wide error, a Bonferroni correction was employed. Thus, LOD scores > 3.36 (*p *= 0.00004, equivalent to a corrected trait-wise LOD > 2) were considered to show experiment-wide suggestive linkage and those with LOD > 4.39 (*p *< 0.000003, equivalent to corrected trait-wise LOD > 3) were considered to have experiment-wide significant linkage.

### Pathway information for clusters showing significant or suggestive evidence for linkage

Information on pathways was assessed using two approaches: 1) using the data provided by GAW15 that were obtained using the publicly available Kyoto Encyclopedia of Genes and Genomes (KEGG) [8] and the Gene Map Annotator and Pathway Profiler (GenMAPP) [9] software and 2) using the Ingenuity Pathways Analysis (IPA) system . KEGG contains large amounts of curated pathway information and GenMAPP is a program that allows visualization and extensive analysis of biological pathways. IPA is a commercially available web-based application that utilizes one of the largest knowledge bases in which information is derived by searching the full text of peer-reviewed publications (as opposed to only abstracts). In addition, using the GenMAPP/KEGG results provided by GAW15, for each cluster showing evidence for significant or suggestive evidence for linkage, we tested whether a pathway was more common among genes within a specific cluster than among genes that were not in the cluster (i.e., the rest of the clusters combined); the statistical significance of the difference was assessed by Fisher's exact test.

## Results

Table 1 gives details of the 29 clusters with heritability ranging from 0.36 to 0.62. The mean (± SD) proportion of variance explained by PC1 for these clusters was 0.46 ± 0.15. The number of genes in a given cluster ranged from 2 to 113. Table 2 shows linkage results for clusters showing LOD > 3.0 with the highest LOD score on chromosome 1 (cluster 67: multipoint LOD = 5.36). In addition, three regions showed evidence for experiment-wide suggestive linkage (i.e., LOD > 3.36). Table 3 shows the pathways represented in the clusters which showed evidence for experiment-wide suggestive and significant linkage using the GenMAPP/KEGG and IPA resources. A majority of pathways were identified by both approaches for clusters 67 and 82, but not for clusters 76 and 93. Furthermore, approximately equal number of genes did not have any pathway information using either IPA or KEGG/GenMAPP (approximately 14, 57, and 35 for clusters 76, 82, and 93, respectively) and the number of genes in a specific pathway ranged from 1 to 6 for a given cluster. This table also shows pathways that were statistically significantly more common among genes within the cluster than among genes that were not in the cluster using the KEGG/GenMAPP data provided by GAW15.

**Table 1:** Clusters with heritability >0.35

Cluster number	Number of genes	Heritability of first principal component
39	38	0.62
82	83	0.55
41	11	0.55
63	42	0.54
27	57	0.52
72	40	0.52
109	10	0.52
76	18	0.52
93	48	0.52
64	24	0.51
59	34	0.51
74	23	0.51
67	2	0.50
5	10	0.50
31	13	0.49
24	2	0.48
29	26	0.46
45	34	0.46
6	35	0.44
7	15	0.44
114	9	0.42
48	32	0.42
43	5	0.42
50	38	0.41
102	60	0.40
62	69	0.39
26	36	0.39
90	110	0.36
111	3	0.36

**Table 2:** Clusters with LOD > 3.0.

Cluster number	LOD score	Chromosome number	Location (cM)
**67**^a^	**5.36**	**1**	**111.71**
93	3.35	3	8.12
48	3.14	3	8.26
93	3.05	7	11.31
39	3.30	7	102.67
**82**	**3.45**	**8**	**140.72**
82	3.11	10	18.80
31	3.14	10	43.72
114	3.14	11	100.02
**93**	**4.06**	**13**	**70.67**
**76**	**3.42**	**14**	**90.25**
82	3.09	16	77.73

^a^Experiment-wide suggestive and significant LOD scores are show in bold font.

**Table 3:** Pathway information for clusters showing evidence of experiment-wide significant and suggestive linkage

Cluster	Number of genes in cluster	Pathway information using KEGG or GenMAPP^a^	Pathway information using the Ingenuity software^b^
67	2	**Glutathione metabolism**^c, d^	**Glutathione metabolism** Xenobiotic metabolism signaling Metabolism of xenobiotics by cytochrome P450
82	83	Smooth muscle contraction ^c^ Circadian exercise ^c^ Fatty acid synthesis^c^ **Valine, leucine, and isoleucine degradation**^c^ **Blood group glycolipid biosynthesis**^c^ **Purine metabolism** Electron transport chain **Oxidative phosphorylation** **G protein signaling** **Apoptosis** mRNA processing reactome	**Valine, leucine, and isoleucine degradation** **Oxidative phosphorylation** **Purine metabolism** Xenobiotic metabolism signaling **G-protein coupled receptor signaling** cAMP-mediated signaling PPAR signaling B Cell receptor signaling **Blood group glycolipid biosynthesis**^e^ **Apoptosis**^e^
93	48	Calcium signaling ^c^ **Cell cycle**^c^ Nucleoside G protein coupling receptor ^c^	**Cell cycle** Protein ubiquitination Intergrin signaling Actin cytoskeleton signaling B cell receptor signaling Leukocyte extravasation signaling
76	18	Apoptosis Inflammatory response^c^	IL-10 signaling

^a^Pathway information using KEGG and GenMAPP were provided in the Problem 1 data set. Pathways with more than one gene are given with the exception of the cluster 76 (inflammatory pathway).

^b^Pathways with more than one gene are given, except for cluster 82, which has 21 pathways with two genes. Information on pathways with ≥ 3 genes are given (with the exception of the blood group glycolipid biosynthesis and apoptosis pathways).

^c^Statistically significantly associated with clusters.

^d^Pathways common to both approaches are given in bold font.

^e^These pathways represent two genes and have been included to show overlap between resources.

## Discussion and conclusion

Several regions of linkage were identified in the analysis of gene expression data from the CEPH families, with the highest LOD score (5.36) observed on chromosome 1 for cluster number 67, composed of two genes: *glutathione S-transferase M1 *(*GSTM1*) and *glutathione S-transferase M2 *(*GSTM2*), both located on chromosome 1p13.3. Individual LOD scores for these regions were 5.33 and 4.53 for *GSTM1 *and *GSTM2*, respectively, which have both been identified in a previous study [2]. To follow up these results, we performed a bivariate linkage analysis of gene expression levels for *GSTM1 *and *GSTM2 *using SOLAR [10]; the LOD score was similar to those obtained for each individual gene expression level. Additionally, we estimated genetic correlation between these two traits, which was significantly different from zero (*p *< 0.0001). This suggests the presence of pleiotropy, i.e., a single gene affecting both traits. The fact that the present methods identify a clear pleiotropic cluster that was identified previously with a different method suggests that linkage results for the larger clusters may also be biologically meaningful. With large data sets, the reproducibility of the results could also be assessed, for example, with k-split methods.

Regions containing clusters 82, 76, and 93 showed LOD > 3.36. Analysis of pathways for these clusters is limited because the majority of genes were not linked to identifiable pathways. Furthermore, significant associations with pathways and clusters could be due to single genes in a pathway in the cluster (e.g., the inflammatory response pathway in cluster 76) or a number of genes sharing a pathway (e.g., six genes that are part of the calcium signaling pathway in cluster 93). At this point we have limited our data to the annotation provided in Problem 1 (i.e., using GenMAPP and KEGG) and building canonical pathways using IPA by providing a set of gene names as input data. While there is overlap between the pathways observed between the two approaches utilized (Table 3), the differences might be due to the source from which pathway information is extracted. Since GenMAPP is a collection of data from voluntary contributions, some bias may be present which might also result in incomplete pathway information. IPA, on the other hand, is an extensive collection of all published literature that is continuously being updated. However, difficulties in interpreting the pathway results may occur because, more than likely, contradicting results will be observed across multiple manuscripts. The researcher will have to proceed with caution when such instances occur.

The present analyses have identified several clusters of multiple transcripts for which the first principal component shows strong evidence of linkage to at least one genomic region. Furthermore, the clustering analysis prior to linkage analysis produces cluster definitions that are defined by the data at hand and do not depend on *a priori *biological knowledge that they constitute a pathway or are genetically co-regulated. Thus, the present approach may identify novel groups of co-regulated transcripts. Many of these clusters contained large numbers of genes (up to 113) and a fully parameterized multivariate analysis would require estimation of a large number of variance components that would be computationally difficult. By conducting analyses of the first principal component, we reduce the transcription information from each cluster into a single variable that maximizes the amount of variance explained. In this exploratory study, we have only used the first principal component for each cluster; a more thorough analysis would include higher principal components, perhaps in conjunction with factor analysis or other multivariable techniques. However, in the present study, PC2 only explained on average 8% of the variance and, therefore, in most cases, PC1 has captured a large part of the information.

A variety of other methods are available for identifying clusters, including hierarchical and machine-learning methods. For the present analyses, we chose non-hierarchical techniques because they are rapid and can assign transcripts to distinct clusters with no assumptions about the nature of the relationships among clusters. All clustering methods are influenced by the scale of the variables included and the present approach employs normalization procedures to equalize the scale among variables. While this approach minimizes the influence of outliers, it would mask the identity of some real clusters, or, perhaps, artifactually introduce some clustering in the data. More research is needed in this area. The present clustering method requires certain assumptions, including specification of the maximum number of clusters. In addition, as presently implemented, the method only identifies clusters in which genes are expressed in the same direction (both up-regulated or both down-regulated). Further work may be required to address these limitations. However, the ability to analyze a large number of traits relatively rapidly and easily shows the advantages of this method. Therefore, the multi-stage method we followed by first performing cluster analysis followed by linkage is another useful approach to identify chromosomal locations for genes affecting expression levels of multiple transcripts.

## Competing interests

The author(s) declare that they have no competing interests.
